# Recent advances in characterizing the physical and functional properties of active packaging films containing pomegranate peel

**DOI:** 10.1016/j.fochx.2024.101416

**Published:** 2024-04-26

**Authors:** Aida Soleimanzadeh, Shabnam Mizani, Ghazal Mirzaei, Elham Taheri Bavarsad, Mehdi Farhoodi, Zahra Esfandiari, Mohammadreza Rostami

**Affiliations:** a Department of Food Science and Technology, National Nutrition and Food Technology Research Institute, Faculty of Nutrition Sciences, Food Science and Technology, Shahid Beheshti University of Medical Sciences, Tehran, Iran; b Department of Food Science, Collage of Agriculture, University of Tabriz, Tabriz, Iran.; c Division of Food Safety and Hygiene, Department of Environmental Health Engineering, School of Public Health, Tehran University of Medical Sciences, Tehran, Iran; d Department of Food Hygiene and Quality Control, Faculty of Veterinary Medicine, University of Tehran, Tehran, Iran; e Nutrition and Food Security Research Center, Department of Food Science and Technology, School of Nutrition and Food Science, Isfahan University of Medical Sciences, Isfahan, Iran; f Food Science and Nutrition Group (FSAN), Universal Scientific Education and Research Network (USERN), Tehran, Iran.

**Keywords:** Active packaging, Fruit by-products, Pomegranate peel, Physical property, Antioxidant property, Antimicrobial property

## Abstract

In recent years, food and packaging industries have worked together to minimize food wastes. Fruit and vegetable by-products, which are known to be among the most abundant food wastes and a great source of bioactive compounds, have the potential to improve food product packaging properties. The antioxidant and antimicrobial properties of pomegranate peel in food active packaging have been the subject of numerous studies. Pomegranate peel has an impact on the films' microstructure and physical properties, such as thickness, water vapor permeability, mechanical properties, optical properties, and thermal properties. Moreover, pomegranate peel incorporated films demonstrate great antioxidant and antimicrobial properties. Reviewing current advancements in the physical and functional properties of active packaging films containing pomegranate peel is the goal of this study.

## Highlights

• Pomegranate peel a major and beneficial by-product in the food processing.
• Antioxidant properties of pomegranate peel extract (PPE) in food packaging.
• Antimicrobial properties of PPE in active food packaging systems.
• Physical properties of films containing pomegranate peel extract.

## 1 Introduction

Active packaging which is developed by intentionally adding certain compounds such as natural extracts into packaging system, is an innovative food packaging concept. In order to enhance the sensory properties of food, ensure food safety, and maintain food quality, this promising technology enables interaction between the packaged food, its packaging, and the interior and exterior environments. It is designed to release active components into the environment surrounding the food or absorb components like oxygen, carbon dioxide, moisture and free radicals that can impair food quality, from the packaged food (Hanani, Yee, & Nor-Khaizura, 2019). Antimicrobial and antioxidant compounds which are considered as active agents, can be added into or on the surface of the packaging material to extend the shelf life of the packaged food (Nur Hanani, Aelma Husna, Nurul Syahida, Nor Khaizura, & Jamilah, 2018). On the other hand, many recent studies have shown that different components of fruits and vegetables like leaves, seeds, peels and unused pulps which are considered as wastes, can be valuable and rich sources of polyphenols, flavonoids, tocopherols, pigments or essential oils which possess several bioactivities, so all of these compounds can be applied in active packaging uses (Kehili, Choura, Zammel, Allouche, & Sayadi, 2018; Putnik et al., 2017; Settanni et al., 2012). The ability of these compounds to enhance the functional properties of packaging materials is the primary reason for the growing interest in the utilization of natural active compounds derived from waste and by-products of the food industry. On the other hand, they are inexpensive sources that eliminate the need for artificial preservatives (Nur Hanani et al., 2018; Yang et al., 2024).

**Fig. 1:**
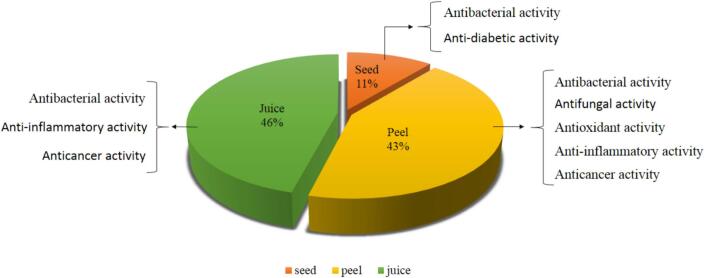
Specific bioactivities linked to pomegranate parts.

**Fig. 2:**
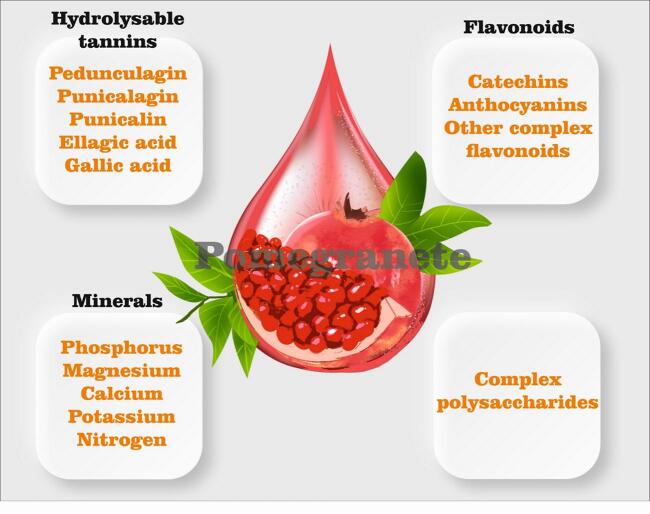
Pomegranate peel bioactive compounds.

*Punica granatum,* also known as pomegranate, is a fruit that originates from the Middle East, India, China, Iran and the Mediterranean regions and has been effective as a natural medicine since 3000 BCE. So it has been considered as an important fruit (Cui, Surendhiran, Li, & Lin, 2020). This blessed fruit which is a member of the punicaceae plant family, has become a functional food all around the world because it is rich in tannins, polyphenols and anthocyanins. Also, it is known as nature's power fruit, which has notable health improving properties. In Fig. 1 the weight percent composition and specific bioactivities of pomegranate different parts is shown. Pomegranate peel makes up about 43% of total fruit weight and is reported to have more antioxidant activity than juice (Charalampia & Koutelidakis, 2017; Pirzadeh et al., 2021). The phytochemical makeup of PPE consists of hydrolyzable tannins, such as punicalin, pedunculagin, and punicalagin, as well as gallic acid (GA) and ellagic acid (EA). These compounds are found in varying amounts ranging from 27 to 172 g/kg. Moreover, PPE is rich in flavonoids, namely catechins, anthocyanins, and other intricate flavonoids. It also includes several organic acids, including citric, ascorbic, malic, fumaric, acetic, tartaric, oxalic, and lactic acids. PPE contains alkaloids such as piperidine and pyrrolidine, minerals including magnesium, nitrogen, phosphorus, potassium, and calcium, as well as complex polysaccharides(Gaharwar et al., 2022). The use of PP has been shown to include bioactive molecules, namely phenolic compounds, that have remarkable biological activity and have the potential to enhance disease indicators. This part is also a major and beneficial by-product in the food processing. Pomegranate peel is an excellent source of compounds like flavonoids, complex polysaccharides, minerals, and hydrolysable tannins which have been shown to be biologically active (Fig. 2). Bioactive parts in pomegranate peel have antibacterial, antioxidant and phenolic activities. It has been confirmed that the main phenolic antioxidant in pomegranate peel is ellagic acid (Firuzi et al., 2019) and has shown a high radical scavenging activity and reducing power (Hayes, Allen, Brunton, O'Grady, & Kerry, 2011). Numerous studies have demonstrated that pomegranate cultivar, fruit part, and level of ripeness all affect the amount and type of polyphenols (Liu et al., 2020). Pomegranate peel extract (PPE) which can be obtained by extraction with many several solvents, also contains polyphenolic compounds that produce significant levels of antioxidant and antimicrobial activity (Ko, Dadmohammadi, & Abbaspourrad, 2021; Maroufi, Tabibiazar, Ghorbani, & Jahanban-Esfahlan, 2021). Prior research has used pomegranate peel extract in edible packaging to prolong the freshness of vegetables and fresh-cut fruits, meat and meat products, dairy, seafood, and bakery food items(Kharchoufi et al., 2018; Khojah, 2020). It should be noted that apart from its uses in food, food packaging, medicine, and cosmetics, PPE may also be helpful in lowering the concentration of heavy metals like arsenite and chromium, as well as basic dyes, in contaminated water. This is because PPE acts as a low-cost bioabsorbent(Andishmand et al., 2023). PP and PPE are considered for all applications due to their antioxidant properties, but one of their most important features is their absence of toxic substances, non-toxicity, and versatility in a variety of food applications, particularly food packaging(Andishmand et al., 2023).

Pomegranate skin is a waste product that humans typically do not consume and is not edible. Due to the presence of many phenolic compounds in this part, which has shown a lot of antibacterial and antioxidant potential, its non-toxicity, cost-effectiveness, and high availability, researchers have used this waste part in active packaging film in many research studies. Different studies have also evaluated the important parameters of a packaging film containing PPE (Vargas-Torrico, Aguilar-Méndez, Ronquillo-de Jesús, Jaime-Fonseca, & von Borries-Medrano, 2024). In this review study, the aim is to explain the use of pomegranate peel extract in packaging films and the effects that this extract has on their antibacterial, antioxidant, and other physicochemical properties.

## 2 Physical properties

### 2.1 Thickness

Because it directly affects the properties of the packaging, such as gas and water vapor permeability, light transmittance, the release of active compounds, and protection against insects and microorganisms, the thickness of the film is regarded as an important physical characteristic of the film (Carvalho et al., 2014; Murmu & Mishra, 2017; Rossi-Márquez, Han, García-Almendárez, Castaño-Tostado, & Regalado-González, 2009). In addition, the uniformity, structure, mechanical strength, and microstructure of the films are influenced by this physical property (Yadav, Kumar, Upadhyay, Pratibha, & Anurag, 2021). Various studies have been performed to examine the thickness of different films containing pomegranate peel.

Liu et al. (2020) produced k-carrageenan films infused with pomegranate peel by utilizing a variety of PPE concentrations (1, 2 and 4 wt% on k-carrageenan basis). The thickness of the film increased as a result of the inclusion of PPE, according to measurements. Because the dispersed extract increased the interstitial spacing between polymeric chains in the film's matrix, the thickness property of the k-carrageenan-PPE films also depended on the amount of extract (Liu et al., 2020). Also, it was proved by Mehdizadeh, Tajik, Langroodi, Molaei, and Mahmoudian (2020) that the thickness of chitosan-starch layers containing PPE increased significantly because the hydrophilic and hydrophobic contents of PPE formed a spongy structure and caused an increase in film thickness (Mehdizadeh et al., 2020). On one hand, Yuan, Lv, Yang, Chen, and Sun (2015) who developed chitosan active films, found that adding 10 g/l PPE and 10 g/l PPE in addition to 10 g/l carvacrol did not notably increase the film thickness (Yuan et al., 2015). Also, application of 10 g/l PPE, and 10 g/l cinnamon essential oil in addition to 10 g/l PPE into the chitosan active films did not cause considerable difference in thickness as observed by Yuan, Lv, Zhang, Sun, and Chen (2016) (Yuan et al., 2016). Nur Hanani et al. (2018) observed that applying 1% pomegranate peel powder (PPP) did not significantly alter the thickness of the gelatin/polyethylene bilayer film, but further enhancement in peel powder concentration notably increased the film thickness. This might have been due to the soluble and insoluble fibers of peel powder which prevent them from fully solubilizing in the gelatin emulsion. Also, SEM images showed that solid particles were placed on the surfaces of active films that contained fruit peels (Nur Hanani et al., 2018). Moghadam, Salami, Mohammadian, Khodadadi, and Emam-Djomeh (2020) claimed that incorporation of pomegranate peel in varying amounts (0, 2.5, 12.5, and 25% *w*/w based on protein weight) into mung bean protein films, resulted in an increase in the thickness of the films. This outcome obtained from an interaction between the functional agents of the biopolymer and the pomegranate peel's phenolic hydroxyl groups, which are capable of increasing the thickness of a film (Moghadam et al., 2020).

### 2.2 Water vapor permeability

Water vapor transmission rate through the food packaging films is a key factor because it directly affects the packaged food safety and shelf life due to its effect on water activity of the food product. Preventing the transfer of water vapor in films used for food packaging is dependent on the film characteristics and external factors including temperature and humidity of the environment. Water vapor permeability (WVP) is expressed as the transmission rate of vapor which pass through a square meter of a flat material of unit thickness. Generally, permeability of the film is influenced by numerous properties of film matrix including the hydrophobicity of the film material as hydrophobic nature of some material reduces the WVP. On the other hand some other structural factors are also important, like presence of cracks or voids, and steric hindrance and tortuosity (Matta, Tavera-Quiroz, & Bertola, 2019).

Yuan et al. (2015) who added carvacrol and PPE (10 g/l of each) to chitosan and produced active films based on chitosan, came to the conclusion that compared to the control sample, addition of 10 g/l PPE alone had no effect on the WVP, but the combination of 10 g/l carvacrol and 10 g/l PPE significantly decreased the WVP of the films. These researchers discovered that chitosan films present high WVP. Therefore, hydrophobic compounds like lipids or essential oils are typically used to reduce WVP (Yuan et al., 2015). Likewise, incorporation of PPE and Tymus kotschanus essential oil (TEO) into the chitosan-starch film was tested: Mehdizadeh et al. (2020) observed that in applying PPE and TEO to chitosan-starch film alone, there were no significant differences in WVP with control, but combination films containing PPE and TEO showed significantly lower water vapor transmission rate. Studies showed that the role of hydrogen groups in creating hydrogen bonds with water can be reduced by hydrogen and covalent bonds between phenolic compounds and the chitosan network, which reduces the tendency of layers toward water. Generally, it has been noted that the WVP values of films are affected by the differences in the strength and the nature of the bonds within the additives (Mehdizadeh et al., 2020). Also, in Hanani et al. (2019) PPP was incorporated into fish gelatin films at concentrations ranging from 1% to 5% (*w*/w based on gelatin weight). The WVP values of these films demonstrated that increasing the proportion of PPP by 1% and 2% produced a remarkable improvement; however, increasing the proportion of PPP by 3% to 5% produced no significant difference in WVP values. SEM analysis revealed that bubbles and incomplete PPP dissolution in gelatin films increase the WVP, which is effective on removing strong bonds that affect the transmission of water vapor through the film. Also, the presence of starch and soluble fibers in pomegranate peel can affect WVP because it has been suggested that the water absorption by gelatin was facilitated by the hygroscopicity of starch under normal atmospheric conditions (Hanani et al., 2019). In a novel approach, Moghadam et al. (2020) created antioxidant edible films with pomegranate peel and mung bean protein that also had PPP concentrations of 0, 2.5, 12.5 and 25% (weight based on dry protein content). There was no significant difference in WVP compared to the control mung bean protein film after adding 2.5% of pomegranate peel extract to the films. On the other hand, adding 12.5 and 25% pomegranate peel to the film dramatically boosted WVP. Due to the high concentrations of pomegranate peel, it seems that certain voids and agglomerated particles were produced on the surface of mung protein films, which caused an increase in WVP (Moghadam et al., 2020). Munir, Hu, Liu, and Xiong (2019) showed that in edible films containing surimi enriched with pomegranate peel extract in different concentrations (2, 4% and 6% protein weight by weight), an increase in the concentration of PPE leads to a decrease in WVP. Several studies proved that hydroxyl groups (OH) as well as the connection between phenolic compounds and proteins can directly affect the WVP of films. (Munir et al., 2019). Hu et al. (2017) observed that adding 1.5% (*w*/w) of PPE to polyethylene (PE) resin resulted in a film with significantly higher water vapor transmission rate than PE film. The reason for this increase can be related to the polarity of polyphenols and water molecules as well as the increase in the gaps between polymer chains formed by PPE aggregation (Hu et al., 2017).

### 2.3 Mechanical properties

The mechanical qualities of edible films refer to their ability to withstand the typical stress that might arise during the transportation and handling of food packed with these films, in order to preserve their integrity and properties. Two main factors in the discussion of mechanical characteristics of food packaging films are tensile strength (TS) and elongation at break (EAB) which generally determine as the film capability to retain the integrity of packaged food products in food supply chain and during storage (Yuan et al., 2015). In fact, TS demonstrates the maximum tensile stress that the film can withstand and reflects the mechanical resistance, while EAB represents the flexibility and maximum resistance of the film to elongation before breaking (Yuan et al., 2016). Film matrix properties, utilized materials, their composition, and intermolecular interactions between them during the preparing process, in addition to preparation conditions play an important role in tensile properties of packaging films (Siracusa et al., 2018; Yadav et al., 2021). Packaging materials should have sufficient strength and stiffness in order to be self-supporting and withstand handling damage (Khalid et al., 2018). As can be seen from Table 1, pomegranate peel has been extensively investigated for this aim.

**Table 1:** Effects of PPE on mechanical properties of manufactured films.

**Film matrices**	**Concentrations of PPE in film forming solution**	**Mechanical effects**	**References**
gelatin-carboxymethylcellulose	150, 300, and 450 mgl^−1^	PPE improved TS and EAB	(Vargas-Torrico et al., 2024)
carboxymethyl cellulose	20 mg/ml	TS was decreased and the EAB was increased by ∼63% and ∼ 25%, respectively	(Khalid et al., 2024)
Polylactic acid- starch	5–20% (w/w%)	PPE improved Young's modulus, TS	(Li et al., 2024)
pomegranate (5% *w*/*v*) and orange peel powders (2% *w*/*v*)	pomegranate (5% w/v) and orange peel powders (2% w/v)	as reducing the particle size the mechanical properties were improved	(Karakuş, Ayhan, & Haskaraca, 2023)
Chitosan/gelatin	1 mg g^−1^	TS increased by 15 mPa	(Bertolo et al., 2022)
starch, poly butylene adipate-*co*-terephthalate	1–3 (g 100 g^−1^)	reduction in TS and increase in EAB	(Flores Fidelis et al., 2022)
polylactic acid	0.5, 1, 1.5 and 2 wt%	Increase EAB and TS decreased	(Dai et al., 2022)
cassava starch	2%, 4%, 6%, and 8% w/w	A minor reduction in mechanical properties	(Esfahani, Mohammadi Nafchi, Baghaei, & Nouri, 2022)
k-carrageenan	1, 2 and 4 wt% on k-carrageenan basis	PPE improved TS and EAB	(Liu et al., 2020)
Chitosan-starch	0.5 and 1% (w/w)	Compared to control, no significant changes in films containing PPE alone combination of PPE and TEO decreased TS and EAB	(Mehdizadeh et al., 2020)
Chitosan	0.01 and 0.03% (w/v)	PPE increased TS and decreased EAB	(Pirsa, Karimi Sani, Pirouzifard, & Erfani, 2020)
Zein	10%	improved the EAB and TS	(Cui et al., 2020)
Polyvinyl alcohol	PPE to SD 1:0, 1:0.5, 1:1, 0.5:1, 0:1	Compared to control, PPE decreased	(He, Lan, Ahmed, Qin, & Liu, 2019)
Surimi	2, 4, and 6% w/w protein content	PPE increased TS and decreased EAB	(Munir et al., 2019)
Polyethylene resin	1.5% w/w	PPE had no significant effect on TS but decreased EAB	(Hu et al., 2017)
Chitosan	10 g/l	PPE alone had no significant effect on TS and EAB. Combination of PPE and CEO increased TS and decreased EAB.	(Yuan et al., 2016)
Chitosan	10 g/l	PPE alone had no significant effect on TS and EAB. Combination of PPE and carvacrol decreased TS and EAB	(Yuan et al., 2015)
Chitosan	1, 2 and 3% (w/v)	PPE decreased TS and increased EAB	(Fan, Zhang, Qin, Zhao, & Cheng, 2013)

PPE: Pomegranate peel extract, TS: Tensile strength, EAB: Elongation at break, TEO: Thymus kotschyanus essential oil, CEO: Cinnamon essential oil, SD: sodium dehydroacetate.

PPE was found to significantly improve the mechanical resistance (tensile strength) and flexibility (elongation at break) of k-carrageenan films when Liu et al. (2020) studied the process of preparing active and intelligent packaging films by adding PPE to the k-carrageenan matrix. As a matter of fact, the abundance of hydroxyl groups led to form the hydrogen bonds between PPE and k-carrageenan chains and increased the compactness of k-carrageenan based films. On the other hand, the dispersed extracts in the film matrix also increase the interstitial spacing between k-carrageenan chains (Liu et al., 2020). Mehdizadeh et al. (2020) reported that the chitosan-starch (CH-S) films including both PPE and thymus kotschyanus essential oil (TEO) showed lower TS and elongation values than CH-S films with no considerable variation in films only containing PPE or TEO, but TS decreased in PPE and TEO treated samples, in the composite film of PPE and CH-S which enriched with TEO for packing beef. The levels of deacetylation and molecular weight of chitosan, affect the TS of chitosan films (Mehdizadeh et al., 2020). Also Fan et al. (2013) who produced an antioxidant chitosan film enriched with PPE (0%, 1%, 2% and 3% (*w*/*v*)), concluded that when the PPE concentration was 1, 2 and 3%, TS decreased about 23%, 32% and 48% respectively. This might be due to the presence of some components like phenolic constituents in PPE likewise hydrophilic properties of that which might have caused the higher EAB and flexibility of the film (Fan et al., 2013). The research carried out by Yuan et al. (2015) showed that incorporating 10 g/l PPE into the chitosan did not result in a significant difference in the TS and EAB of the chitosan films. This was probably due to the interaction between the chitosan matrix and the phenolic compounds found in pomegranate peel. However, the TS and EAB of chitosan films were significantly reduced when 10 g/l PPE was combined with 10 g/l carvacrol. These modifications may have occurred as a result of the film's incorporation of hydrophobic agents, which resulted in a structure with decreased mobility, flexibility, and fracture resistance (Yuan et al., 2015). In another study, Yuan et al. (2016) carried out an experiment by addition of cinnamon essential oil (CEO) and PPE in to the chitosan film. The results showed that adding 10 g/l PPE did not cause significant changes in TS and E% but incorporation of 10 g/l CEO with 10 g/l PPE caused a notable enhancement and reduction in TS and E% of chitosan films, respectively. The presence of CEO, which has a strong interaction with the polymer and reduces the polymer's free volume and molecular mobility, could be the cause of these changes in TS and EAB. (Yuan et al., 2016). Pirsa et al. (2020) who created a biodegradable antibacterial film from chitosan (CS)/PPE and *Melissa officinalis* Essences (MOE), concluded that the TS increased when PPE and MOE were added to the chitosan films. Among all the films, CS/PPE0.03%/MOE0.0% and CS/PPE0.03%/MOE0.5% had the highest TS respectively (Pirsa et al., 2020). The uniform dispersion of PPEs within the polymer matrix may be the outcome of this improvement in TS. Chitosan and PPEs interact strongly through ion bonding, and chitosan/essential oil and pomegranate extracts can form new, strong bonds (Dehnad, Mirzaei, Emam-Djomeh, Jafari, & Dadashi, 2014). PPE and MOE contain numerous compounds, including steroid compounds, monoterpenes, amino acids, polyphenols, and phenolic aldehydes. Some links in the structure of the film are created by these compounds, which have a high molecular weight. As a result, the film's softening decreases while its resistance to tensile stress increases (Pirsa et al., 2020). Also, the humidity of film decreases because of the hydrophobic character of MOE and PPE which leads to increase to tensile stress and tear (Dehnad et al., 2014). It has been shown that the chitosan film has the highest EAB and flexibility but adding MOE and PPE decreases the EAB, may be due to the uniform distribution of the PPE (Pirsa et al., 2020). He et al. (2019) used electrospinning to incorporate PPE and sodium dehydroacetate (SD) into an active film made of polyvinyl alcohol (PVA). He et al. then investigated how the ratio of PPE to SD affected the mechanical properties in a consistent total mass fraction of 5% in the film-forming solution. The PVA film without any additive had the highest TS of 10.38 MPa and an EAB of 48.44%, according to the findings. The mechanical properties were weakened by the high PPE content (up to 5%). Similar effect was also exhibited by SD content. It was found that the addition of PPE and SD altered the neatness of the PVA film molecular organization. The incorporation of PPE decreased the TS and increased the EAB, resulting in a flexible and stretchy film. Adding SD to the PPE containing film (sample ratio 1:1), significantly reduced EAB compared with the PVA film only containing PPE (sample ratio 0:1) and applying PPE and SD to the PVA film together improved the TS significantly (when the PPE to SD ratio was 1:1). Briefly, PPE and SD showed a synergistic effect on the mechanical properties of the PVA film and films containing both PPE and SD (sample ratios 0.5:1, 1:1, 1:0.5) showed an increase in TS and a decrease in EAB, which indicates their synergistic effect (Davar, Majedi, & Mirzaei, 2018; He et al., 2019). Also, Munir et al. (2019) prepared silver carp surimi-based edible films in which various amounts (2%, 4%, and 6% *w*/w protein content) of pomegranate peel under acidic conditions were incorporated. The results indicated that the film incorporated with PPE showed higher TS and lower EAB than the control film (Munir et al., 2019). Covalent and non-covalent bonding between the phenolic compounds in films containing phenolic compounds, led to the films with improved rigidity (Prodpran, Benjakul, & Phatcharat, 2012; Rattaya, Benjakul, & Prodpran, 2009). On the other side, incorporation of extracts lead to greater rigidity of films due to the reduced plasticizer effects through the interactions between surimi proteins and phenolic compounds (Li, Sinclair, & Li, 2011). Additionally, EAB values were reduced by increasing concentrations of PPE, thus it can be concluded that the phenolic compounds affected the TS and EAB of manufactured films, relating to the source and concentration of PPE (Munir et al., 2019). Hu et al. (2017) observed that the incorporation of 1.5% (*w*/w) PPE into the polyethylene (PE), significantly increased EAB. This could be related to the presence of low molecular weight compounds in pomegranate peel that penetrate the space created by the amorphous phase of the polymer structure and act as plasticizing agent. Polymer-polymer interactions were weakened following the increase in PPE level and penetration of the PPE agent into the crystalline region. These changes resulted in destroying the crystalline structure of the film. On the other side, little effect on the TS of the polyethylene film was observed by the incorporation of 1.5% PPE (Han, 2005; Hu et al., 2017).

### 2.4 Optical properties

The color parameter and transparency can affect product appeal, appearance, and consumer acceptance and is therefore an important feature of the film (Jridi et al., 2019; Kumar, Neeraj, & A., & Singh, R., 2019; Ojagh, Rezaei, Razavi, & Hosseini, 2010). Moreover, reducing light transmission is desirable in order to protect and preserve food, meanwhile UV light leads to destructive effects like nutrient losses, off-flavor, and discoloration, which are often caused by lipid oxidation. Various additives have ability to change the film primary color and transparency by linking to the structural composition of them. Moreover, it has been proven that the type and concentration of extracts directly affect the color, as well as it has been suggested that light transmission varies according to the variety and quantity of phenolic compounds (Moradi et al., 2012; Munir et al., 2019; Zhang et al., 2024).

Yuan et al. (2016) investigated that how CEO and PPE affected the color and transparency of chitosan films. When CEO and PPE were added to chitosan films, the films' L* (lightness/darkness) values notably decreased, but their a* (redness/greenness) and b* (yellowness/blueness) values increased in comparison to control samples. This could be because the peel of pomegranates contains polyphenols. (Yuan et al., 2015; Yuan et al., 2016). Also, the transparency of the films with the addition of PPE and combination of CEO and PPE was remarkably reduced comparing with the control sample, which can be related to polyphenols existence in the films (Yuan et al., 2015). In a study by Moghadam et al. (2020), the opacity and color properties of films made from mung bean protein that contained pomegranate peel were assessed. The findings demonstrated that pomegranate peel integration raised the films' a* and b* values as well as their opacity. While the brightness (L* value) dropped with increasing pomegranate peel content in the matrix of film samples, these parameters increased noticeably. These alterations in the mung bean protein film's darkness, yellowness, and redness can be attributed to the anthocyanins which are found in the pomegranate peel (Moghadam et al., 2020). Kumar et al. (2019) who prepared chitosan-pullulan blended edible films enriched with PPE, observed that the incorporation of PPE had no effect on transparency property of developed edible films (Kumar et al., 2019). Munir et al. (2019) reported that the lowest L* values were related to the silver carp surimi-based edible films containing pomegranate peel. Moreover, this parameter decreased with increasing the concentration of PPE. However, the b* values increased with the concentration of PPE. The high color pigment concentrations in PPE led to an increase in the b* value of the films with increased PPE content, and the pigments effectively reduce light transmission. PPE-enriched surimi films had less transparency than the control film, and it got worse as the PPE concentration increased. (Munir et al., 2019). In a recent investigation, He et al. (2019) came to the conclusion that PPE considerably decreased the lightness (L*) of all PVA films, and that increasing the extract's concentration causes the lightness to drop. Due to the electrospinning technique, PVA-based films had very low transparency, and adding PPE to the films had no noticeable impact on that transparency regardless of the concentration of PPE (He et al., 2019).

### 2.5 Thermal properties

In food packaging films, thermal properties which indicate their ability to withstand decomposition at high temperatures, determines the melting temperature of the film (Zhang et al., 2019). Common techniques which are used in measuring this important characteristic are thermogravimetric analysis (TGA) and differential scanning calorimetry (DSC) (Yong & Liu, 2020). In the TGA technique, the weight changing is represented as a function of temperature or time in the form of thermo-grams (Piyada, Waranyou, & Thawien, 2013), while a DSC measurement can determine the temperature profile and energy changes during continuous heating and cooling. Also, crystallization temperature (Tc), melting temperature (Tm), and glass transition temperature (Tg) can be found by this technique (Jafarzadeh & Jafari, 2021).

The thermal properties of zein/CSNPs/PPE nanocomposite film and neat zein film were examined in a study by Cui et al. (2020), and it was discovered that the thermal deterioration of neat zein film occurred in two stages. Due to the loss of water and other volatile substances from the zein film, the first stage occurred at a temperature between 100 and 150 degrees Celsius. A drastic weight loss was evident at temperature between 200 and 250 degrees Celsius, which was attributable to the thermal breakdown of the primary protein components in the zein film. In comparison with thermal breakdown of the neat zein film, the addition of the CSNPs/PPE improved the thermal stability of the nanocomposite film. At nearly 200 °C, the first weight loss occurred, and at 300 °C, the second. Furthermore, DSC was used to examine the thermal behavior of the zein/CSNPs/PPE nanocomposite film and neat zein film. A large endothermic peak (Tg) in the region of 50–150 °C was found in the zein film. (Cui et al., 2020). Luís, Domingues, and Ramos (2019) claim that, the evaporation of volatile substances like water is determined by the presence of these peaks. The TGA is in line with this result. In addition, the highest endothermic peak was observed at 271 °C. Due to the intermolecular interactions between CSNPs and zein molecules, which improve the thermal stability of the nanocomposite film, the heat flow steadily increased after the CSNPs/PPE matrix was added (Luís et al., 2019; Zhang et al., 2019). Munir et al. (2019) investigated the effect of PPE on thermal properties of silver carp surimi-based edible films. All films (control film and PPE incorporated films containing 2, 4, and 6% *w*/w extract) indicated four weight loss stages. All films experienced weight loss during the first stage between 140.2 °C and 150.2 °C. The film's ability to absorb both free and bound water led to weight loss in this range. Additionally, films containing PPE lost less weight than control films. This may be because surimi films contain less water because PPE phenolic compounds are more hydrophobic. Proteins which have low molecular weight and structurally bound water and glycerol (a plasticizer) loss were directly associated with surimi film's second stage weight loss, which occurred between 200.1 and 215.3 °C. (Hoque, Benjakul, & Prodpran, 2011; Munir et al., 2019). Weight loss and thermal degradation temperature values if films containing PPE at different concentrations, were less than control films. The film's lower glycerol content as a result of the extract's inclusion may be the cause of this decrease in weight loss. The next stage weight loss appeared about onset of 310.3 °C- 350.5 °C for all surimi films. This was attributed to degradation or breakdown of higher interacting protein portions. At the final stage, weight loss of the films was seen between 425.6 °C and 455.3 °C. This phase was possibly linked to loss of compounds that are stable at high temperatures. Furthermore, Weight loss and thermal degradation temperature were enhanced in incorporated films and it was suggested that the stability of the films can be affected by the addition of PPE. at high temperature because of phenol-protein interactions which led to a stable and compact microstructure resulting in thermal stability improvement of incorporated films (Wu et al., 2013). Following the DSC test, Kumar et al. (2019) observed that adding different concentrations of PPE to the chitosan-based edible films, reduced the glass transition temperature of the films and the highest glass transition temperature was for the pure chitosan film. Also, the findings demonstrated that the thermal stability of the film samples, which is dependent on the glass transition temperature, decreases with the increase in the amount of PPE. (Kumar et al., 2019).

## 3 Microstructure properties

The microstructure of the films which is frequently characterized using scanning electron microscopy (SEM), can determine the arrangement of the various components of the film and help to better understand the film physical characteristics. Generally, a compact and homogenous structure which is free from voids and cracks, is an advantage for mechanical and physical characteristics of the films (Yong & Liu, 2020).

The microstructures of zein film, zein/CSNPs/PPE nanocomposite film, and plasma-treated zein/CSNPs/PPE nanocomposite film were recently examined using SEM by Cui et al. (2020). The nanocomposite film produced fine dispersion of the CSNPs/PPE matrix on the surface of the film, whereas the zein film had a plane surface structure. However, the plasma treatment's etching effect completely modified and roughened the surface of the zein/CSNPs/PPE nanocomposite film. (Cui et al., 2020). Ali et al. (2019) conducted another study in which revealed that the surface morphology of starch films incorporated with PPP concentrations of 2% and 10% showed a smooth surface, while PPP marks were clearly visible on the surface of the starch matrix. Additionally, no gap was seen between the starch matrix and PPP particles, which represented good compatibility. Moreover, higher concentrations of pomegranate particles were found to cause their agglomeration in the starch matrix (Ali et al., 2019). Moghadam et al. (2020) observed the images of the surface and the cross-section of mung bean protein films filled with 0, 2.5, 12.5, and 25% of pomegranate peel and revealed that there was a continuous microstructure with no void and crack in the surface areas of any film samples. The surface morphology of the control mung bean protein film was uniform and smooth, and the addition of pomegranate peel at a concentration of 2.5% did not cause any significant change in this surface morphology. However, films containing high concentrations of pomegranate peel had more heterogeneous microstructure with more permeability toward moisture and were determined by observing white dots on the surface of the films. The undissolved particles of pomegranate peel incorporated in the films might cause white spots on the surface of the films. Cross-section images of all films showed compressed and uniform structure with no pore. In control films, unlike films enriched with pomegranate peel, some layers were detected. So, films formed in the presence of pomegranate peel have more compact structure which could be caused by the formation of intermolecular forces (like hydrogen bonds and hydrophobic interactions) between the protein chains and the bioactive molecules present in the pomegranate peel (Moghadam et al., 2020). According to Liu et al. (2020) the surface of k-carrageenan film was homogenous and plain, indicating the film-forming components were suitable and phase segregation did not happen, while the incorporation of PPE markedly enhanced the surface roughness of k-carrageenan film. Also, the results showed that the surface roughness increased at higher concentrations of PPE (Liu et al., 2020).

## 4 Antioxidant activity

**Fig. 3:**
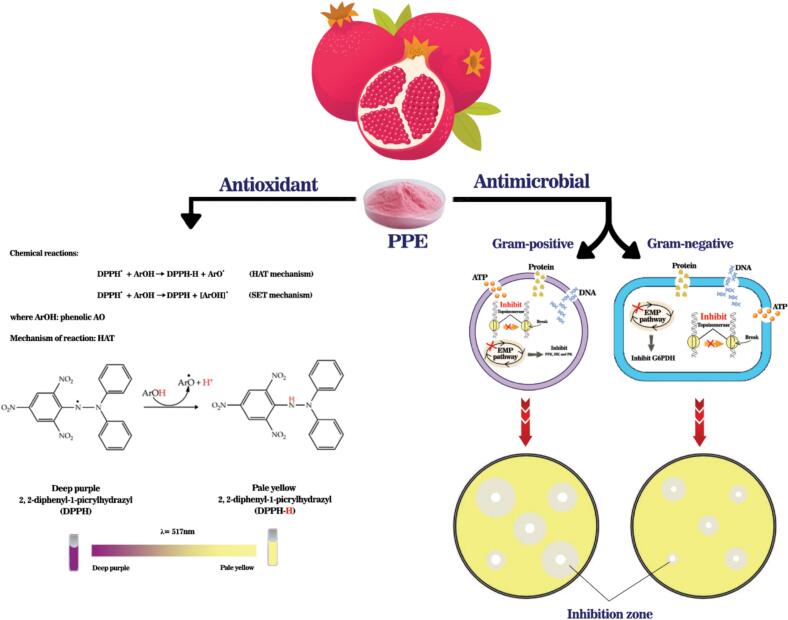
Antioxidant and antimicrobial properties of PPE.

One of the essential chemical elements which is very important in the metabolism of aerobic organisms is oxygen, but it can also cause adverse reactions. Free radicals, which are reactive oxygen species (ROS), often react with different molecules such as proteins, lipids, DNA, and RNA and can damage them(Fig. 3) (Augustyniak et al., 2010; Carocho & Ferreira, 2013; Lobo, Patil, Phatak, & Chandra, 2010; Lü, Lin, Yao, & Chen, 2010; Valko et al., 2007; Valko, Rhodes, Moncol, Izakovic, & Mazur, 2006; Xiong et al., 2022; Xiong et al., 2022). They can also cause diseases such as cancer (Valavanidis, Vlachogianni, Fiotakis, & Loridas, 2013; Valko et al., 2006), arthritis (Hadjigogos, 2003), diabetes, arthrosclerosis (Rajendran et al., 2014), neurodegenerative diseases (Wojtunik-Kulesza, Oniszczuk, Oniszczuk, & Waksmundzka-Hajnos, 2016), and premature aging (Getoff, 2007). In addition to the human body, oxidation reactions occur in many food products that are exposed to oxygen, light, or heat, leading to reduced shelf-life and nutritional value of products, as well as changes in their taste and color. Antioxidants can protect cells by various mechanisms such as forming non-radical species from ROS and reducing oxygen concentration, thus being very important in maintaining the quality of products (Carocho, Barreiro, Morales, & Ferreira, 2014; Dorman, Peltoketo, Hiltunen, & Tikkanen, 2003; Jayaprakasha, Singh, & Sakariah, 2001; Oroian & Escriche, 2015). Food products are not available to consumers for consumption immediately after production; rather this process takes time and these products must maintain their original quality and be safe when consumed (Caleja et al., 2015a). Since oxidation affects product quality, delaying the oxidation of biomolecules by antioxidants is an effective strategy in maintaining product quality and can be used as a protective mechanism in food storage (Peschel et al., 2006). Synthetic antioxidants such as tert-butyl hydroquinone (TBHQ), propyl gallate (PG), butylated hydroxytoluene (BHT), and butylated hydroxyanisole (BHA) have been used excessively in the food industry due to their higher stability and performance, low cost, and availability (Saad et al., 2007; Xiu-Qin, Chao, Yan-Yan, Min-Li, & Xiao-Gang, 2009). However, recent studies have shown that their long-term use can cause skin allergies, gastrointestinal tract problems, cancer (Bleve et al., 2008; Botterweck, Verhagen, Goldbohm, Kleinjans, & van den Brandt, 2000; Engin, Bukan, Kurukahvecioglu, Memis, & Engin, 2011; Jeong, Kim, Kang, Ku, & Cho, 2005; Randhawa & Bahna, 2009), premature aging, and DNA damage (Kornienko et al., 2019). Natural antioxidants can be good alternatives to synthetic antioxidants. They are mostly extracted from plant materials such as vegetables, spices, fruits, and herbs (Bansal et al., 2013; Dimitrios, 2006; Jiang & Xiong, 2016); they are classified into three main categories: phenolic compounds, carotenoids, and vitamins (Dorman et al., 2003; Elżbieta, Cieslik, & Topolska, 2008; Jayaprakasha et al., 2001). Some studies have reported that non-edible parts of fruits often have higher bioactive contents than edible parts (da Silva, Nogueira, Duzzioni, & Barrozo, 2013; Freitas et al., 2015; George, Kaur, Khurdiya, & Kapoor, 2004; Gorinstein et al., 2001). Pomegranate is a fruit rich in antioxidants; with each ton of pomegranate juice producing 9 tons of by-products that are a good source of functional compounds (Diamanti, Igoumenidis, Mourtzinos, Yannakopoulou, & Karathanos, 2017). Pomegranate peel, which is considered as an agricultural waste, is a great source of bioactive compounds and has several health benefits due to its polyphenolic components such as tannins and flavonoids, anthocyanins, alkaloids, and organic acids (Malviya, Arvind, Jha, & Hettiarachchy, 2014). These natural antioxidants, which are found in various parts of the plants including leaves, roots, stems, fruits, seeds and peels, should be extracted for further uses (Shah, Bosco, & Mir, 2014). The most common method is extraction with organic solvents (Azmir et al., 2013; Naczk & Shahidi, 2004). Other methods such as extraction with supercritical fluids, microwaves, high hydrostatic pressure, and ultrasound can also be employed (Oroian & Escriche, 2015).

Utilization of antioxidants in the food industry as preservatives can be in food matrices, food packaging coatings, and films (Caleja et al., 2015b; López de Dicastillo, Rodríguez, Guarda, & Galotto, 2016; Wang & Gao, 2013). High amounts of oxygen transfer in the packaging cause oxidation and affect the taste, color, as well as texture of the food product. As a solution, antioxidant active packaging can remove oxygen from the head space or release antioxidants into the package. This type of active packages can be in the form of independent antioxidant devices (sachets, pads, or labels with antioxidant agents) or antioxidant packaging materials which can be placed into the walls of the films or inside the product container (Fang, Zhao, Warner, & Johnson, 2017; Gómez-Estaca, López-de-Dicastillo, Hernández-Muñoz, Catalá, & Gavara, 2014). To measure the antioxidant capacity, the oxidation rate of chemicals such as DPPH (2,2′-diphenyl-2-picrylhydrazyl), TBARS (2-thiobarbituric acid substances), AAPH (2,2′-azobis(2-methylpropionamidine) dihydrochloride), and ABTS (2,2′-azino-bis(3-ethylbenzothiazoline-6-sulfonic acid)) which are exposed to antioxidants is determined (López-Alarcón & Denicola, 2013). According to the points mentioned above, it is important to prevent oxidation of food products where pomegranate peel extract (PPE) have been demonstrated to be a good source of antioxidants and can be used in active packaging.

Table 2 reports the results of recent studies on antioxidant properties of biopolymer films containing PPE. Liu et al. (2020) compared the antioxidant activity of k-carrageenan-pomegranate flesh extract (PFE) and k-carrageenan-PPE films via DPPH radical scavenging method. K-carrageenan film revealed the lowest DPPH radical scavenging activity due to its low hydrogen donation ability. Adding PFE or PPE to the films significantly enhanced their DPPH radical scavenging activity. At the same amount of extract addition, films containing PPE showed significantly higher DPPH radical scavenging activity than those containing PFE due to higher total phenol in PPE (Liu et al., 2020). Similar conclusions were reached by Gull et al. (2021) who added PPE to nanochitosan coatings and investigated the antioxidant activity of apricots. Apricots treated with chitosan and PPE had higher DPPH radical scavenging activity compared to the control sample. The low antioxidant activity of control sample was because of fruit senescence and higher respiration or degradation of phenolic compounds (Ghasemnezhad, Shiri, & Sanavi, 2010). Fruits coated with chitosan and PPE indicated the highest antioxidant activity, which can happen for these reasons; barrier properties of coatings and modifying internal atmosphere or scavenging free radicals as well as chelating metals by phenolic compounds of PPE (Gull et al., 2021). The effect of adding different concentrations of pomegranate peel on the characteristics of mung bean protein films were investigated by Moghadam et al. (2020). Pomegranate peel contains phenolic compounds (catechins, punicalin, pedunculagin, punicalagin, gallic acid, and ellagic acid) resulting in production films with a higher total phenol content compared to the control sample (Smaoui et al., 2019). The results revealed a significant improvement in the antiradical activity and reducing power of the films by elevating the concentration of pomegranate peel thus increasing the amount of total phenol (Moghadam et al., 2020).

**Table 2:** Effect of PPE on antioxidant activity of manufactured films.

**Type of Biopolymer Matrix**	**Concentration of PPE**	**Food Applications**	**Antioxidant Activity**	**References**
gelatin-carboxymethylcellulose	150, 300, and 450 mgl^−1^	raspberry fruit	82.76% to 89% DPPH	(Vargas-Torrico et al., 2024)
jackfruit seed starch	0.02, 0.04, 0.06, 0.08, and 0.1 g/mL	prolonging the shelf life of white grapes	87.35 ± 1.64% DPPH	(Bodana et al., 2024)
Carboxymethyl cellulose/gelatin	0, 0.5, 1, 1.5, and 2% *v*/*w*	shelf life of beef up to 3 days.	84.15 ± 0.12% DPPH	(Nabeel Ahmad, Yong, Wang, Munawar, & Zhu, 2024)
The pomegranate (5% w/v) and orange peel powders (2% *w*/*v*)	The pomegranate (5% w/v) and orange peel powders (2% w/v)	food packaging	97% DPPH	(Karakuş et al., 2023)
low-density polyethylene	500, 1000, 5000, 10,000, 15,000, and 20,000 ppm	active food packaging	48.46 to 74.43 ± 2.50% DPPH	(F, A, G, Z, & N, 2023)
chitosan, gelatin	1 mg g^−1^	strawberries preservation	–	(Bertolo et al., 2023)
polylactic acid	0.5, 1, 1.5 and 2 wt%	Food Packaging	96.2 ± 0.8% and 93.1 ± 0.5% based on DPPH and ABTS	(Dai et al., 2022)
polyvinyl alcohol	79.4 PPE (mg GAE)	Food Packaging	74.82 ± 0.18% DPPH	(Saroha, Khan, Raghuvanshi, & Dutt, 2022)
3% w/v of cassava starch	2%, 4%, 6%, and 8% w/w	monitoring lamb meat freshness	70% DPPH	(Esfahani et al., 2022)
Cress seed gum chitosan nanoparticles	chitosan: PPE ratio of 1: 0.50 (w/w)	Food Packaging	23.3–69.9% DPPH	(Soltanzadeh et al., 2022)
Taro starch-casein composite	(0, 10, 25, 50, 100 wt%)	Food Packaging	80.66% for DPPH, 56.81% ABTS, and 33.84 mg GAE/g of film for FRAP	(More, Pegu, & Arya, 2022)
chitosan/gelatin	0.5–5.0 mg g^−1^	photoinactivation of bacteria	56.90 ± 3.17% DPPH	(Dias et al., 2022)
Chitosan-Pullulan	5%	Tomato	from∼20% to∼28% (after 15 days 23 ͦ C DPPH) from∼30% to∼36% (after 15 days 4 ͦ C DPPH)	(Kumar et al., 2021)
Tuna skin collagen-chitosan	0.5 g/L	–	50–60% DPPH	(Qu, Xiong, Wang, Li, & Zhang, 2022)
Nanochitosan	0.5, 0.75 and 1% (w/v)	Apricot fruit	from∼38% to∼47% (after 30 days DPPH)	(Gull et al., 2021)
k-carrageenan	1, 2 and 4 wt%	–	from∼18% to∼50% PPE (DPPH) from∼15 to∼38% PFE(DPPH)	(Liu et al., 2020)
Mung bean	0, 2.5, 12.5 and 25% (w/w)	–	from∼5% to∼65% (DPPH) from∼29% to∼98% (ABTS)	(Moghadam et al., 2020)
Polyvinyl alcohol	5% (different ratios of PPE and SD)	–	from∼2% to∼48% (DPPH)	(He et al., 2019)
Fish gelatin	1–5% (w/w)	active packaging	from∼59.74% to∼71.82% (DPP) from∼48.40% to∼80.02% (ABTS)	(Hanani et al., 2019)
Fish gelatin/polyethylene	0–9% (w/v) fruit peels	–	from∼74.5% to∼79% PPE (DPPH) from∼1.5 μg/g to∼4 μg/g PPE (ABTS)	(Nur Hanani et al., 2018)
Chitosan	0, 1, 2 and 4% (w/v)	Rainbow trout	from∼0.17 mg/kg to∼1.2 mg/kg (TBARS) from∼0.17 mg/kg to∼0.69 mg/kg (TBARS)	(Berizi et al., 2018)
Zein	0, 25, 50 and 75 mg g^−1^	Himalayan cheese	from∼40% to∼80% (DPPH)	(Mushtaq et al., 2018)

DPPH: 2,2′-diphenyl-2-picrylhydrazyl, ABTS: 2,2′-azino-bis (3-ethylbenzothiazoline-6-sulfonic acid), SD: sodium dehydroacetate,TBARS: 2-thiobarbituric acid substances, ABTS: 2,2′-azino-bis (3-ethylbenzothiazoline-6-sulfonic acid).

Elsewhere, He et al. (2019) prepared active composite films of polyvinyl alcohol with different ratios of PPE and sodium dehydroacetate via electrospinning. The antioxidant activity of films was measured by DPPH method. According to the results, pure polyvinyl alcohol films and polyvinyl alcohol films with sodium dehydroacetate had the lowest antioxidant activity, while the films containing PPE had a higher antioxidant activity (He et al., 2019). Enhanced antioxidant activity is mainly influenced by polyphenols. Polyphenolic structures of PPE are polar hydroxyl groups and punicalagin. Punicalagin is a high molecular weight polyphenol of pomegranate peel (Nasiriboroumand, Montazer, & Barani, 2018) and its high antioxidant activity is due to 16 dissociable O—H groups (Kaderides, Papaoikonomou, Serafim, & Goula, 2019; Shin et al., 2017; Zhuang et al., 2019). Kumar, Neeraj, and Trajkovska Petkoska (2021) added PPE to chitosan-pullulan edible coating and studied its effect on the quality and shelf-life of tomato for 18 days at 23 and 4 °C. The total phenol content of the samples and as a result their antioxidant activity diminished, while the storage time increased. The chitosan-pullulan coating containing PPE retained the phenolic compounds of the tomato during storage and significantly controlled the reduction of antioxidant activity in the treated samples (Kumar et al., 2021). This was due to the coating's ability to delay phenol oxidation and enzymatic activity, as well as to reduce ethylene production and respiration rate (Dong, Cheng, Tan, Zheng, & Jiang, 2004). As a new packaging, zein films with different concentrations of PPE were prepared for packing Himalayan cheese (Kalari/kradi) by Mushtaq, Gani, Gani, Punoo, and Masoodi (2018). The study showed the control films that did not contain PPE had a low phenol content and lacked antioxidant activity, but the films containing the extract revealed a higher phenol content and antioxidant properties, which rose with increasing the amount of extract. The results indicated that there were interactions between extract's polyphenols and zein, where polyphenols such as ellagic acid, punicalagin, and gallagic acid caused antioxidant activities in the films. Thus, the use of these films in Himalayan cheese packaging significantly delayed the oxidation reactions during the storage period (Mushtaq et al., 2018).

Berizi, Hosseinzadeh, Shekarforoush, and Barbieri (2018) incorporated PPE into chitosan coatings and prepared coated rainbow trout. They observed that the samples subjected to chitosan and different concentrations of PPE had less peroxide and TBARS values during storage than the control sample. PPE contains phenolic compounds, proanthocyanidins, and flavonoids, so it has antioxidant effect and can inhibit superoxide hydroxyl and peroxyl, which are effective in the oxidation of fats (Berizi et al., 2018). Chitosan reduces lipid oxidation through chelating ferrous ions and preventing peroxide activities of ferric ions (Fan et al., 2009). In Berizi's study, chitosan with 4% loading level of PPE acted as an antioxidant during 6 months of fish storage (Berizi et al., 2018). Another study by Hanani et al. (2019) examined the effect of pomegranate, papaya, and jackfruit peel powder on properties of gelatin/polyethylene films. They noticed that the total phenol content of the films increased significantly with the addition of 1% fruit peel powder where pomegranate caused the highest amounts of total phenol content in the films (Nur Hanani et al., 2018). The antioxidant property of the films was due to the presence of large amounts of polyphenols in the pomegranate peel (Emam-Djomeh, Moghaddam, & Yasini Ardakani, 2015; Yang, Lee, Won, & Song, 2016). Also, by inhibiting free radicals and chelating metals, it could reduce oxidation and delay the quality deterioration of the product (Contini et al., 2014). DPPH levels increased in the films containing the extract, which indicates the antioxidant activity of the film. Also, the control films had DPPH radical scavenging activity, which could be due to the presence of gelatin and its antioxidant activity, since amino acids such as phenylalanine, tyrosine, and tryptophan can react with radicals by their phenolic side chain (Wang, Hu, Ma, & Wang, 2016). Further, Hanani et al. (2019) used pomegranate peel powder (PPP) in films containing fish gelatin and examined its various properties, including the antioxidant property of the films. The antioxidant activity of the films was determined by DPPH and ABTS methods. The anti-radical activity was significantly enhanced in the films containing PPP, with the highest antioxidant activity found in the films containing 5% PPP (Hanani et al., 2019). The reason is the presence of biologically active compounds such as phenolic compounds, anthocyanins, and punicalagin, which have the good potential to scavenge free radicals and chelate metals (Kalaycıoğlu & Erim, 2017). Control films had also antioxidant activity, which could be due to antioxidant peptides in fish protein (Kavoosi, Shakiba, Ghorbani, Dadfar, & Mohammadi Purfard, 2015).

## 5 Antimicrobial activity

Antimicrobial compounds are synthetic or natural compounds that are used to inhibit the growth of spoilage or pathogenic microorganisms. The presence of these compounds leads to the safety and quality improvement in food products. In recent years, various studies have been conducted to extract and utilize plant compounds as agents to prevent food-borne diseases and to prevent spoilage in food. Plant compounds are usually safe and easily decomposed, and do not harm the environment. Many fruits and vegetables have potential chemical compounds that can act as antimicrobial agents in food (Singh, Singh, Kaur, & Singh, 2019). According to Fig. 3, pomegranate is one of the fruits that with its greatest antimicrobial effect found on its peel extract (Alexandre et al., 2019). The antimicrobial effect of pomegranate peel is related to the chemical compounds within its structure, including polyphenolic compounds such as flavonoids, phenolic acids and tannins (like punicalagins, gallic acid, alginic acid), which have a synergistic effect with each other (Dey et al., 2012). Following terpenoids, polyphenols are the largest group of secondary plant compounds and have aromatic rings containing hydroxyl groups (Singh et al., 2016). Polyphenols are linked with proteins in bacterial cell walls, which break down the cell wall structure. They also disrupt bacterial metabolism through combination with sulfhydryl groups of soluble proteins (Akhtar, Ismail, Fraternale, & Sestili, 2015). The hydroxyl groups in polyphenols also lower the pH around the bacterial cell membrane and disrupts the process of bacterial metabolism ultimately causing the death of bacteria (Pisoschi et al., 2018). According to the studies of Vaquero & De Nadra (2008), it was concluded that some flavanols from the group of flavonoids such as quercetin in PPE can enhance cell membrane permeability in bacteria such as *Escherichia coli* and affect the production of ATP. They interfere with the growth and multiplication of bacteria. Tannins also reduce the adhesion of microbial cells and decrease the mineral consumption of bacterial cells forming complexes with bacterial polysaccharides and inhibiting bacterial growth (Asadishad, Hidalgo, & Tufenkji, 2012).

**Fig. 4:**
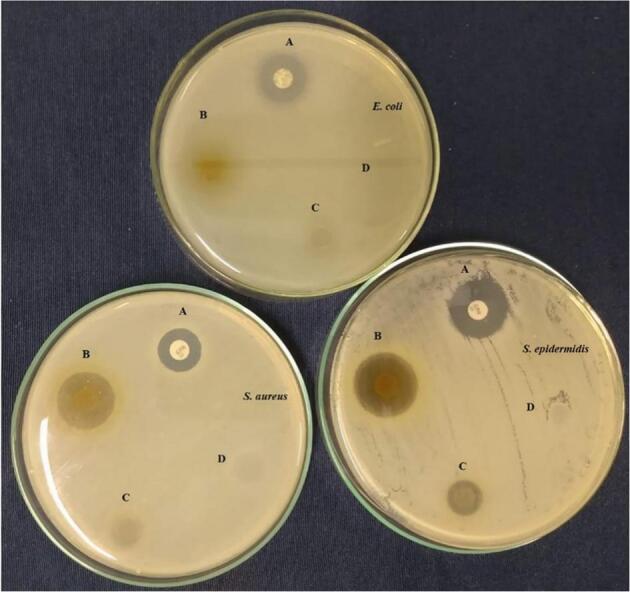
The provided photographs depict the disc diffusion test conducted on strains of *E. coli*, *S. aureus*, and *S. epidermidis*. The images represent the following conditions: a positive control using ampicillin (A), a film containing PPE at a concentration of 1.25% *w*/*v* (B), a film containing PPE at a concentration of 0.25% w/v (C), and a film without PPE (D)(Costa et al., 2020).

Costa et al.*(*Costa et al., *2020**)* was conducted to make films using a combination of poly(vinyl alcohol), starch, poly(acrylic acid), and PPE. PPE is a bioactive chemical known for its antibacterial and healing properties, making it suitable for use as a bioactive wound dressing. The minimal inhibitory concentration (MIC) of the PPE was first examined using an in vitro methodology. Antimicrobial activity was shown in films with a lower concentration of PPE against both *Staphylococcus aureus* and *Staphylococcus epidermidis*(Fig. 4***)***.

In a study, crude pomegranate peel extract was used to prevent bacterial proliferation. *Staphylococcus aureus*, *Bacillus subtilis* and *Pseudomonas aeruginosa* were inhibited at concentrations of 0.062 and 0.25 mg/ ml of peel extract respectively (Foss et al., 2014). Another study evaluated the antibacterial properties of PPE. Based on the obtained results, this compound is able to prevent the growth of a wide range of bacteria, specially gram-positive ones such as *Bacillus subtilis* and *Staphylococcus aureus*, and gram-negative ones like *Escherichia coli* and *Klebsiella pneumoniae*. PPE was used at concentrations of 0.2 to 0.78 mg / ml / l, and all bacteria were inhibited (Fawole, Makunga, & Opara, 2012). Yuan et al. (2015) studied the effect of carvacrol and PPE on chitosan-based films. Regarding antimicrobial properties, the films containing carvacrol revealed a good antimicrobial activity, but the addition of 10 g / l of PPE in combination with 10 g / l of carvacrol significantly enhanced the antimicrobial resistance of the film, presenting the greatest effect against Gram -positive bacteria such as *Staphylococcus aureus* and Gram -negative bacteria such as *Escherichia coli* (Yuan et al., 2015). Gram-negative bacteria are usually less sensitive to antimicrobial compounds than Gram-positive bacteria. This difference is due to the structure of the wall of the cell since its contains Gram-negative lipopolysaccharide bacteria that can prevents antimicrobial groups from affecting the cell cytoplasm (Ouattara, Simard, Holley, Piette, & Bégin, 1997). In another study, the properties of fish gelatin-based films with PPE were investigated. *Staphylococcus aureus*, *Listeria monocytogenes*, and *Escherichia coli* were evaluated for antimicrobial properties. Adding 1% of PPE into the films with the base of gelatin had no effect on antimicrobial properties, but upon elevating the concentration to 2% and 5%, the antimicrobial properties increased significantly. The greatest antimicrobial effect was on *Staphylococcus aureus* bacteria; so this study proves the greatest antimicrobial effect of PPE is on Gram-positive bacteria (Hanani et al., 2019). Emam-Djomeh et al. (2015) performed a study to evaluate the properties of the film based on sodium caseinate containing pomegranate peel extract. Here, the antimicrobial aspects of *Escherichia coli* and *Staphylococcus aureus* were tested as two Gram-negative and positive bacteria. The antimicrobial effect of PPE on gram-positive bacteria was proven where 125 to 25 however, 0 ppm of this compound in the film could inhibit the growth of *Staphylococcus aureus*; however, to inhibit the growth of *Escherichia coli*, to 250 up to 500 ppm of this compound was used (Emam-Djomeh et al., 2015). The antimicrobial and antioxidant potential of PPE to determine the shelf-life in chicken products was evaluated and compared by Kanatt, Chander, and Sharma (2010). PPE indicated acceptable antioxidant activity in terms of antioxidants, but the seed extract had no significant antioxidant properties. At an inhibitory concentration of 0.01% of PPE, this compound showed high antimicrobial activity against *Staphylococcus aureus* and *Bacillus cereus*. Pseudomonas was inhibited at concentrations above 0.1%, but this amount of extract did not show antimicrobial effect against *Escherichia coli* and Staphylococcus typhimurium. PPE prevented spoilage due to oxidative acidity in poultry products and thus increased food safety (Kanatt et al., 2010). Mushtaq et al. (2018) evaluated the packaging of Himalayan cheese (kalari) with a zein film containing different concentrations of PPE, and measured different characteristics of this packaging in their experiment. The antimicrobial properties of the films were also evaluated with the bacteria studied being *Escherichia coli*, Pseudomonas perfringens, *Micrococcus luteus*, Enterococci faecalis, *Staphylococcus aureus*, *Proteus vulgaris*, and *Salmonella typhi*. The control sample that no PPE. PPE showed antibiotic properties which was dose dependent, where three different concentrations of 25, 50, and 75 mg of PPE were added to the films. The antimicrobial effect of the film increased with elevating the concentration of the extract where at a concentration of 75 mg all bacteria were completely destroyed (Mushtaq et al., 2018). In a similar study exploring the properties of zein films containing catechins and gallic acid on the shelf-life of fresh cheeses, the same antimicrobial results were obtained confirming the antimicrobial properties of chemical compounds in PPE, including gallic acid (Ünalan, Arcan, Korel, & Yemenicioğlu, 2013). In a study, fungal chitosan (Ch) obtained from Aspergillus niger and PPE were used to prepare the coating layers of Oreochromis niloti-cus fillet where their microbiological and, chemical properties as well as sensory quality were evaluated. The fillets were stored at 4 °C for one month. Fish fillets with films containing 2% chitosan with concentrations of 0.5, 1, 1.5, and 2% of PPE were coated. Growth of total aerobic microbes, Psychotropic bacteria, Enterobacteriaceae, coliforms, Salmonella, *E. coli*, yeast, and mold as well as *Staphylococcus aureus* was observed during storage in the refrigerator (at 4 °C) for 30 days. In control samples without chitosan coating and PPE, the number of bacteria increased over time. Adding chitosan to the fish coat reduced the number of bacterial colonies, but adding PPE to chitosan significantly lowered the number of bacteria. Almost all studied bacteria were killed and this effect increased with raising the concentration of PPE from 1 to 2%. The most sensitive bacterias to chitosan films and PPE were Salmonella and *Staphylococcus aureus*. Coating fish fillets with Ch and Ch in addition to PPE can be used as a natural preservative to boost the shelf life and maintain microbial safety (Alsaggaf, Moussa, & Tayel, 2017).

In addition to bacteria, fungi and their mycotoxins can also lead to food spoilage and endanger the health of the consumer while also causing massive economic damage. PPE, as a natural compound can act as an antifungal agent. High amounts of polyphenolic compounds in this extract have antifungal effects. Tannins also inhibit fungal growth and high levels of punicalagin in pomegranate peel also present antifungal activity (Tehranifar, Selahvarzi, Kharrazi, & Bakhsh, 2011). In a study, the antibacterial and antifungal activity of PPE, seed extract, pomegranate juice, and whole fruit was investigated on selected bacteria and fungi. The Gram-positive bacteria evaluated included *B. coagulans*, *B. cereus B. subtilis* and *S. aureus*, while Gram -negative bacteria were *E. coli*, *K. pneumoniae*, and *P. aeruginosa*. The evaluated fungi were *A. niger* P. citrinum, *R. oryzae*, T. reesei, and *M. indicus*. The results indicated that pomegranate peel had the highest antimicrobial activity than other parts of the fruit. Among selected bacterial and fungal cultures, the highest antibacterial activity was recorded against Gram-positive *Staphylococcus aureus*, while among fungi, high activity was recorded against Aspergillus Niger (Dahham, Ali, Tabassum, & Khan, 2010). In another experiment, Nair, Saxena, and Kaur (2018) examined the effect of PPE on enhancing the shelf-life and preventing the growth of Colletotrichum gloeosporioides in Capsicum. PPE at a concentration of 1% was used in preparing two films based on chitosan and alginate, with the antimicrobial properties also investigated. When the PPE was used alone as a treatment, it inhibited the growth of C. gloeosporioides radial mycelium up to 100%, and this property was also proven in combination with chitosan plus alginate films. When the chitosan-based films was used alone, it could significantly (*P* < 0.05) inhibit mycelial growth (68.8%) compared to alginate treatment (12.9%). Laboratory results showed that the incorporation of 1% PPE to chitosan and alginate coating solutions in the preparation of edible coating could significantly boost their antifungal activity against the growth of C. gloeosporioides up to 100% in Capsicum (Nair et al., 2018).

## 6 Conclusion and future perspective

Increasing demands for consumption safe food products had led to emergence of developed packaging methods. Packaging is one of the main processes in food production that is effective in maintaining food quality and safety, prolonging shelf-life and enhancing its organoleptic properties. Active packaging using functional compounds of plants, is a new packaging technology. PPE contains large amounts of phenolic and bioactive substances that have functional properties such as antioxidant and antimicrobial properties. This has been proven many times in numerous studies. The presence of these substances has led to the use of this extract in applications related to human health as supplements and therapeutic properties, as well as use in food science, including food packaging. PPE increases the shelf life of meat, dairy products, fruits, etc. For this reason, this combination has been used many times as an effective ingredient in the composition of food packaging films or coatings. This review summarizes the effect of using PPE as a bioactive compound on the antioxidant, antimicrobial, microstructure and physical properties including thickness, WVP, mechanical properties, optical properties and thermal properties of packaging films. Results of the research indicate that produced films' properties have been affected by PPE and have been improved in some cases.

Pomegranate peel is a waste that is not normally consumed by humans. The analyzes carried out by the researchers have shown that the pomegranate peel waste has materials with suitable properties for use in food packaging. PPE has shown high potential in the production of packaging films, both in physical and mechanical properties, as well as in antioxidant and antimicrobial properties. Considering that this material is a waste and the cost of its preparation is low, and it also has the capability of a conversion process, its use in the food industry, especially in food packaging, to maximize the advantageous properties of pomegranate peel in relation to emerging trends in active edible packaging and sustainable food processing technology, it is imperative that future research concentrates on ascertaining the most favorable levels or proportions of pomegranate peel extract, as well as its potential synergistic effects with different edible matrices. The use of this material on an industrial scale for this purpose and also to reduce the produced waste should also be considered. In addition, via the reduction of biowaste items within supply chains, the need for landfill disposal will be reduced.

## CRediT authorship contribution statement

**Aida Soleimanzadeh:** Writing – original draft. **Shabnam Mizani:** Investigation. **Ghazal Mirzaei:** Writing – original draft. **Elham Taheri Bavarsad:** Conceptualization. **Mehdi Farhoodi:** Writing – review & editing. **Zahra Esfandiari:** Writing – review & editing. **Mohammadreza Rostami:** Writing – review & editing, Methodology, Conceptualization.

## Funding

This study has not been funded by any institute.

## Authors' contributions

All authors contributed to investigation, conceptualization, analysis, and were involved in the writing process.

## Declaration of competing interest

The authors declare that they have no competing interests

## Data Availability

No data was used for the research described in the article.
